# The impact of high intensity care around birth on long-term neurodevelopmental outcomes

**DOI:** 10.1186/s13561-020-00279-8

**Published:** 2020-07-08

**Authors:** Corneliu Bolbocean, Michael Shevell

**Affiliations:** 1 Department of Preventive Medicine, University of Tennessee Health Science Centre, 66 N. Pauline Street, Memphis, TN 38163 USA; 2 The Centre for Addiction and Mental Health, Toronto, Ontario, 33 Russell St, Toronto, ON M5S 2S1 Canada; 3 Department of Pediatrics, Faculty of Medicine, McGill University, 3605 Rue de la Montagne, Montréal, QC, H3G 2M1 Canada

**Keywords:** Neonatal care, Hospital intensity, Hospital costs, Long-term outcomes

## Abstract

**Background:**

An equitable and affordable healthcare system requires a constant search for the optimal way to deliver increasingly expensive neonatal care. Therefore, evaluating the impact of hospital intensity around birth on long-term health outcomes is necessary if we are to assess the value of high intensity neonatal care against its costs.

**Methods:**

This study exploits uneven geographical distribution of high intensity birth hospitals across Canada to generate comparisons across similar Cerebral Palsy (CP) related births treated at hospitals with different intensities. We employ a rich dataset from the Canadian Multi-Regional CP Registry (CCPR) and instrumental variables related to the mother’s location of residence around birth.

**Results:**

We find that differences in hospitals’ intensities are not associated with differences in clinically relevant, long-term CP health outcomes.

**Conclusions:**

Our results suggest that existing matching mechanism of births to hospitals within large metropolitan areas could be improved by early detection of high risk births and subsequent referral of these births to high intensity birthing centers. Substantial hospitalization costs might be averted to Canadian healthcare system ($16 million with a 95% CI of $6,131,184 - $24,103,478) if CP related births were assigned to low intensity hospitals and subsequently transferred if necessary to high intensity hospitals.

## Introduction

Hospital costs are a major driver of overall healthcare expenditures, especially in countries with a universal healthcare system where access to expensive health facilities is virtually unrestricted, but essentially geographically circumscribed due to high travel costs. Rising hospitalization costs related to neonatal care is particularly a growing concern for policymakers in developed countries [1–3]. Although advances in neonatal care are linked to gains in survival and other meaningful health outcomes, the high costs associated with birthing hospitals requires decision makers to explore methods that optimize neonatal healthcare delivery.

Balancing the fundamental tradeoff between increasing costs of new technological advances and of neonatal care and ability to save lives of the majority, even the sickest and smallest newborns has become a challenge for healthcare systems in North America. *Perinatal regionalization*—the tiered provision of neonatal care—has emerged over time as a strategy to balance this fundamental tradeoff. This approach provides optimal risk-appropriate maternal child services for geographically dispersed populations. These systems of regional tiered perinatal services, now common across North America and around the world, are linked to improved outcomes for high-risk infants born either preterm or with serious medical or surgical conditions [4–6].

Regionalization of perinatal care has facilitated the diffusion of newly-developed neonatal technologies and has improved access of newborns in the community to innovative interventions including therapeutic hypothermia. The impact of improved neonatal interventions in early childhood on later life outcomes is documented and continues to grow in economics literature on the topic. Specifically, Bharadwaj et al. show that children who receive extra medical care at birth have lower mortality rates and later in life achieved higher test scores and better grades in school [7]. Conversely, Figlio et al. argue that the effects of poor neonatal health on adult outcomes are pervasive [8]. Cutler and Meara evaluated the care for low birth weight infants and their results ultimately indicated that medical spending for aggressive care at birth is worthwhile [9]. The extant clinical and health research demonstrates the links between perinatal regionalization with improved neonatal outcomes for infants born preterm and with low birth weight [10].

Although research shows that early health interventions lead to improvements, there is mounting concern regarding the high costs of neonatal intensive care alongside the substantial financial burden survivors of neonatal intensive care might pose on their families and healthcare system [3, 11]. While the overall efficacy of specific advances in neonatal medicine has been established in the literature [12–14], limited evidence currently exists on the overall effectiveness of technological change in neonatal care; particularly regarding its impact on long-term health outcomes.

This study examines the effect of hospital intensity around birth on the long-term health outcomes by analyzing data drawn from the CCPR. The level of care at birth hospitals directly impacts the type of therapy a newborn receives immediately following their birth. Thus, level of care is critical to treat any potentially adverse events during labor and delivery and it determines the later severity of CP. As such, we evaluated the relationship between hospital type around birth (high intensity, Level III or Level II hospital vs low intensity, or Level I hospital) and CP non-ambulatory status.

The challenge in examining the relationship between hospital type and CP non-ambulatory status is the level of care available around birth is not randomly assigned. Thus, hospital type (high intensity vs low intensity) at birth is our potentially endogenous regressor. We aimed to correct for selection bias which originates as a result of high-risk pregnancies and births more likely taking place in high intensity hospitals (Level III, II). To remove the effects of selection bias on estimates we exploit uneven geographical distribution of high intensity hospitals across Canada, and unique etiology of CP. In particular, we instrument the choice of birth hospital using instrumental variables related to the mother’s location of residence around birth (indicators for central metropolitan areas and physical distance from mother’s residence at birth to the closest high intensity hospital). The basic intuition here is that within the Canadian universal healthcare system the mother’s location of residence around birth provides plausibly exogenous variation in hospital choice. Moreover, proximity determines travel costs and therefore the access to high intensity hospitals for a geographically dispersed population. This enables us to compare births and associated outcomes assigned to hospitals with different treatment intensities.

We focus on children diagnosed with CP for two specific reasons. First, our exclusion restriction comes from the etiology of the condition, particularly the mother’s location of residence around birth, as this is orthogonal to all currently known CP biological determinants (we provide empirical evidence for this statement). According to Mosalli [15] the severity of the condition could be lessen if any form of treatment such as access to therapeutic hypothermia were available. However, that this treatment is only available at Canadian high intensity hospitals, and can be clinically effective within the first six hours of life. Moreover, infants are not anymore eligible for cooling if therapeutic hypothermia cannot be initiated after the critical window of the first 6 h of life. Second, our dataset includes all of the currently known risk factors associated with CP which in turn allows us to control for essentially all known physiological pathways to exclude an alternative explanation. We further used resampling methods to test the hypothesis that the distribution of clinical, social and economic covariates is consistent with random assignment with respect to the mother’s residence at birth (rural vs urban).

We have two primary findings. First, we found robust evidence that birth at high intensity hospitals does not lessen eventual CP severity, despite the fact that only these types of hospitals have clinically effective technology related to the outcome. This finding means that differences in the clinically effective technology across hospitals are not associated at the margin with the severity of the condition. We performed a resampling analysis to test the validity of our instruments and the hypothesis that children in our dataset have the same distribution of covariates across rural and urban areas. Second, we found that the distribution of a group of covariates between urban and rural areas did not differ significantly from 1000 random reassignments of existing patients between rural and urban areas. This suggest that the assignment of CP related births to types of hospitals is essentially random. Our results indicate that there is no gain found in increasing the assignment of CP related births to high intensity hospitals.

Our findings suggest that substantial healthcare costs could be averted within the Canadian healthcare system if CP related births were assigned to low intensity hospitals and subsequently transferred if necessary to high intensity hospitals. We question the conventional wisdom that treatment at high intensity hospitals results, by default, into more effective long-term health outcomes. Our estimates suggest that the Canadian healthcare system could save around $16 million per year in hospitalization costs under the scenario where CP related births were first assigned to less intensive hospitals and subsequently transferred to high intensity hospitals if necessary (for example after an event of intrapartum fetal distress).

The study proceeds as follows. Section 1 provides an outline of relevant medical background, and presents the unique features of the CP etiologic mechanism. Details regarding our data sources along with sample inclusion and exclusion criteria are provided in Section 2. Section 3 begins by outlining our empirical framework and details the estimation strategy. In this section we also report our main estimates and explore the robustness of these results. We present the results of cost-benefit analysis in section 4, offer discussions in Section 5 and conclude in Section 6.

## Background

### Cerebral palsy

CP is a set of variable heterogeneous clinical symptoms which result from either an anomaly and/or acquired injuries to the motor regions of the brain [16], causing graded levels of observable neuro-motor dysfunction [17]. CP remains the largest single cause of childhood physical disability in the developed world [18] and it is estimated to affect approximately 2.5–4.0 infants per 1000 live births [19–23].

Although onset occurs at birth or in early childhood, CP persists throughout an individual’s life leaving patients to incur a significant economic burden. In 2003, the U.S. Center for Disease Control (CDC) and Prevention estimated the lifetime costs of CP per individual to be $921,000 USD [24]. A more recent Danish study from 2009, reports the approximate lifetime cost of CP was €860,000 for men and €800,000 for women. The largest component of these expenditures was social care costs, particularly during childhood [25]; however, the lifetime costs of CP depends on the condition’s severity and associated comorbid conditions (e.g. intellectual disability, epilepsy, etc.).

CP is often linked to intrapartum events specifically intrapartum hypoxia [26]. Allegations of obstetrical clinical negligence are commonly cited as a cause of CP and usually focus on the obstetrical care provided in the intrapartum period [27, 28]. We are aware of only one study which explored the relationship between the quality of care given to a mother during labor and delivery and later CP. An association between suboptimal care and CP was found in a small proportion of CP cases [29]. However, previous research did not control for the level of immediate neonatal and postnatal care available following labor and delivery. The relationship between the level of neonatal care available at birth, perinatal and neonatal factors, and the severity of CP is not yet presently known.

### Cerebral palsy and intrapartum events – a narrow window of opportunity

Acquired brain injuries during labor and delivery, such as perinatal asphyxia, can cause cerebral palsy. Perinatal asphyxia affects 3–5 infants per 1000 live births, with 0.5–1 infants per 1000 live births developing brain damage in the form of neonatal encephalopathy (NE) [30]. Evidence of at least two of the following indicates presence of intrapartum hypoxia: Apgar score of 5 or less at 10 min; need for mechanical ventilation; metabolic or mixed acidosis, or any infant blood gas within the first hour of life showing a pH of 7 or less or a base deficit of ≥16 mmol/l [15].

The level of care at the birth hospital plays a crucial role in reducing the disability after an acquired brain injury. Consistent standards of level of neonatal care across Quebec, Canada, indicate that the standard of care is available for NE such as: mechanical ventilation and access to therapeutic hypothermia is available at hospitals with NICUs [15, 31]. In a typical childbirth the baby born in a nursery who suffers from fetal distress is resuscitated and transferred for additional care to Level III hospital. The benefit of neonatal intensive care, however, may only be clinically effective within a narrow window of opportunity and during the first hours of life. For example, infants are no longer eligible for cooling if therapeutic hypothermia cannot be initiated within the first 6 h of life [15].

### Perinatal regionalization

The concept of perinatal regionalization emerged in North America, first in Canada [32], followed in the U.S. in 1971 by the American Medical Association’s House of Delegates’ report [33]. Birth hospitals were classified into one of three levels based on the degree of complexity of maternal and perinatal care each was capable of providing. European countries implemented a decentralized maternity services in order to ensure good access to necessary care independent of a mother’s place of residence [34]. Moreover, recent research have shown that type of hospital is not the main determinant of hospital financial performance in Europe [35].

There is a large body of clinical literature that demonstrates how high-risk infants have better health outcomes in delivery hospitals with neonatal care. In a comprehensive review of 41 published studies conducted between 1979 and 2008, involving the use of different research designs Lasswell et al. [10] concluded: “for very low-birth-weight and very preterm infants, birth outside of a Level III hospital is significantly associated with an increased likelihood of neonatal or predischarge death.”.

Due to its size and relatively sparse population, Canada has a highly regionalized neonatal-perinatal care system and nearly all births take place in public hospitals. Nurseries (birthing centers and Level I care) are designed to accommodate the high-number of low-risk births, while specialty care (Level II) and subspecialty care (Level III) accommodate the needs of medium and high-risk births. This classification denotes the differences in the level of perinatal resources and obstetric competence available at a specific birth hospital.

### Provider incentives

Healthcare providers in Canada do not have the financial incentives to refer patients to high or low intensity hospitals. Moreover, there are no clinical guidelines in Canada for a referral to a high vs low intensity birth hospital. This implies that geographic accessibility is the primary determinant of choice of maternity care providers in Canada [36] like in European countries with a universal healthcare system [37]. We thus use mother’s residence at birth to instrument for level of care at the birth hospital.

## Material and methods

### Data

The study was conducted using Quebec provincial data from the CCPR as the level of neonatal care at birthing center was available only for this province. Children with CP born in 1999 or later were enrolled within six of the province’s 17 administrative health regions, capturing approximately half of the province’s population within the CCPR. Parental consent is obtained and maternal medical and obstetric records, as well as the child’s neonatal, medical, and rehabilitation records, are reviewed. These data are supplemented by a standardized parental interview and physical examination of the child by a pediatric neurologist, developmental pediatrician, or child physiatrist. For each enrolled child, more than 120 variables are collected and entered into a Research Electronic Database Capture database. To be enrolled in CCPR, a child must be at least 2 years of age and meet diagnostic criteria for CP, including a clinical diagnosis of a non-progressive motor impairment resulting from a presumably early injury to the developing brain [17].

Children within the CCPR included for analysis in this study were born between 1999 and 2014 in the province of Quebec. Children with CP diagnosis linked to any identified post-neonatal cause or cases born outside the province of Quebec were excluded from our investigation.

For our analysis, we classified children according to the level of neonatal care available at birth hospital [38]. In Quebec maternity care is regionalized and nearly all deliveries take place in public hospitals or birthing centers.^1^ This classification reflects differences in the level of perinatal resources and obstetric competence available at a specific birth hospital. We used clear, uniform definitions and consistent standards to classify the level of neonatal care across the study sites, and made appropriate adjustments for differences in case mix between the three groups of hospitals. We classified each hospital delivery unit according to its level of neonatal care using the policy statement on this topic provided by the American Academy of Pediatrics [38] (Appendix). This classification reflects differences in the level of obstetric and neonatal competences available at the birth hospital, and is outlined in further detail in Appendix.

In brief, nurseries are designed to provide care for newborns 34 weeks gestation or more, and can offer intravenous therapy, phototherapy and gavage feeding; Level II centers provide care for newborns 30 weeks gestation or more, and in addition to level I services can offer non-invasive ventilation or endotracheal intubation. Level III centers care for newborns regardless of gestational age and in addition to the above services offer nitric oxide therapy, therapeutic hypothermia along with immediate access to a complete range of pediatric subspecialties, imaging, and surgeries.

The outcome used for this analysis was CP non-ambulatory status, as defined by a Gross Motor Function Classification System (GMFCS) level IV or level V [40]. The major challenge for our research was to control for case-mix differences between types of hospitals. In particular, Level II and Level III hospitals have a higher proportion of medium and high-risk pregnancies compared with Level I hospitals for obvious reasons. We used a quasi-experimental study design [41], controlling for relevant covariates in order to control for selection bias that could originate from the differences in case mix between the three groups of hospitals. Our rich dataset allowed us to control for all known risk factors related to etiology of CP.

We used current clinical practice guidelines in obstetrics and gynecology [42], perinatal surveillance literature [43], CP risk factors [44] and clinical judgment to choose explanatory variables and to make proper adjustments for differences in case-mix between different delivery hospitals. The following covariates were used to control for possible selection bias: birth weight, gestational age, preeclampsia, gestational diabetes, bleeding during pregnancy, severe illness during pregnancy, accident or trauma during pregnancy, preterm birth, a family history of CP, low maternal education (lacking a high school diploma), maternal age, and history of drug use. We also controlled for perinatal asphyxia, which was defined as neonatal encephalopathy with at least three of the following criteria: an Apgar score < 5 at 10 min, a cord pH of < 7.0, a cord base excess > 16, an abnormal fetal heart rate such as tachycardia (> 160 beats per minute) or bradycardia (< 120 beats per minute), presence of meconium, need for intubation, delay in spontaneous respiration, need for resuscitation of the newborn, or abnormal imaging results consistent with hypoxic ischemic injury.

Our cohort of 825 children with CP without any post-neonatal cause were born in Quebec between 1999 and 2014. Forty-seven percent were born in birth sites with Level III neonatal care, 21% with Level II and the remainder (31%) with Level I neonatal care. Non-ambulatory status (Gross Motor Function Classification System level IV and level V) was reported in 27% of the cases. The other characteristics of the sample are presented in Table 1.

**Table 1:** General Characteristics of the Population

Variables	Children with CP (*N* = 825)
**Level of Service at Delivery**	
Level I (%)	31
Level II (%)	21.6
Level III (%)	47.4
**Non-Ambulatory Status (GMFCS IV-V) (%)**	26.7
**Maternal Age,** mean ± SD	29.64 ± 5.6
**Mother’s ethnic group**	
Caucasian (%)	78.76
**Education**	
High school or more education (%)	78.06
Less than high school education (%)	21.94
**Family History of CP** (%)	4.14
**Resuscitation at birth** (%)	38.25
**Type of Pregnancy**	
Single foetus (%)	89.2
**Pre-eclampsia** (%)	7.7
**Gestational Diabetes** (%)	11.4
**Bleeding during first trimester of pregnancy** (%)	18.9
**Severe Illness during Pregnancy** (%)	28
**Accident or Trauma during Pregnancy** (%)	13
**Birth weight (gram),** mean ± SD	2645 ± 1007. 1, 663
**Gestational age (weeks),** mean ± SD	35.73 ± 4.9663
**Prematurity (< 37 weeks)** (%)	46.8
**Perinatal Asphyxia** (%)	12.04

## Empirical strategy

### Overview

Our empirical objective was to isolate the causal effect of hospital intensity (high intensity - level III or level II vs low intensity level I) available at birth on the probability of being diagnosed with the least severe Cerebral Palsy type. The challenge in examining this research question is that the level of neonatal care at birth is not randomly assigned. We therefore offer an identification strategy that does not rely on random assignment. We employ resampling techniques to test the hypothesis that covariate distributions across the mother’s residence at time of birth (rural and urban areas) are consistent with random assignment.

### Implementation

#### Instrumental variables and generalized method of moments

We used multivariate instrumental variables regression along with generalized method of moments, which allows for unobserved risk factors that affect the choice of hospital type and outcomes conditional on that choice, but which often suffers from imprecise estimates. Instrumental variables estimation uses additional covariates that influence the choice of birth hospital but which do not influence CP severity.

Geographic accessibility is one of the most significant determinants of the type of maternity care providers in Canada [36] as in European countries with a universal healthcare system [37]. We thus use mother’s residence at birth to instrument for level of care at birth hospital. In particular, pregnant women residing within census metropolitan areas are more likely to give birth in hospitals with level II, or level III neonatal care as these types of hospitals are located in large metropolitan areas. However, the type of residence is orthogonal to unobserved components of CP non-ambulatory status as given that the type of residence (rural vs, urban) is not systematically related to clinical pathways and etiology of this neurological disease. Because of this, the mothers’ residence at birth should in theory be a good statistical instrument. We further used resampling methods to test the hypothesis that *the distribution of clinical, social and economic covariates is consistent with random assignment with respect to mothers` residence at birth (rural* vs. *urban).*^2^

In the first stage of estimation, we predicted the type of hospital at birth using exogenous indicators for greater Montreal, Quebec City, Gatineau, Sherbrooke, or an indicator for a census metropolitan area. We used Statistics Canada classification of census metropolitan areas to construct a central metropolitan area indicator as well as Population and Dwellings Counts for Canada.

$$ {D}_i={\pi}_0+{\pi}_1{Z}_i+{X}_i^{\prime}\pi +{\varepsilon}_i. $$

Our primary equation of interest was the second stage, in which we regress CP non-ambulatory status on the predicted probability of hospital type $\hat{D_i}$ and other covariates:

$$ {Y}_i={\beta}_0+{\beta}_1\hat{D_i}+{X}_i^{\prime}\gamma +{e}_i $$

In order for our estimation approach to deliver consistent parameter estimates, the instruments *Z*_*i*_ from our first stage must not directly affect CP non-ambulatory status and be uncorrelated with the unexplained variation of CP non-ambulatory status. The evidence presented in Table 4 indicates our instruments are very strong and relevant.

Maternal residence at birth may not always be a perfect predictor for the level of neonatal care at delivery hospitals, especially in large metropolitan areas. In Montreal for example, a pregnant woman can choose either level of NICU. However, we believe that mothers’ residence at birth is the strongest determinant of the birthing hospital given the peculiarities of a universal healthcare system in Canada. Thus, ceteris paribus, a pregnant woman is more likely to choose a delivery hospital with the highest available NICU in the area, which is Level III, or Level II in metropolitan areas.

#### Endogenous bivariate Probit model

We also estimated this model using a bi-variate probit regression, where both the type of hospital at birth, *D*_*i*_, and CP non-ambulatory status, *Y*_*i*_, are estimated as functions of the standard normal probability distribution function, P[.]. The first stage can be written:

$$ {D}_i=P\Big[{\pi}_0+{\pi}_1{Z}_i+{X}_i^{\prime}\pi +{\varepsilon}_j $$],

and the outcome is determined by:

$$ {Y}_i=P\left[{\beta}_0+{\beta}_1{D}_i+{X}_i^{\prime}\gamma +{e}_i\right]. $$

The correlation between *e*_*i*_, *ε*_*i*_ is the source of omitted variable bias. The identification requires that excluded instruments *Z*_*i*_ be independent of *e*_*i*_, *ε*_*i*_, which are assumed to be normally distributed. Given the distributional assumptions imposed on error terms this model can be estimated using maximum likelihood estimation.

#### Resampling

*For our results to be a credible estimate of the effect of hospital type on CP non-ambulatory status, it was necessary to rule out the possibility that children born with CP in rural areas differ systematically from children born with CP in urban areas, lest differences in CP non-ambulatory status be caused by these differences instead of differences in birth hospital type. To test for this, we compared the distributions of urban* versus *rural statistical characteristics to distributions derived of these same summary statistics where existing children were randomly assigned to urban and rural residences. If the statistical characteristics of urban and rural CP cases are statistically indistinguishable, the proportion of Mann-Whitney difference of means tests using randomly assigned residences that are less than the Mann-Whitney test for actual urban* vs. *rural residences (empirical p-value) will be uniformly distributed from 0 to 1. A test of whether a group of empirical p-values representing multiple statistical attributes of CP cases is uniformly distributed is an effective test of whether the statistical attributes of urban* versus *rural CP cases differ systematically. (Good, 2006; Carrell and West, 2009).*

## Results

Table 2 contains the results of our resampling analysis. In the first column, we tested the differences in urban versus rural means of the 17 health covariates listed in the notes to Table 2. We failed to reject the null hypothesis of a uniform distribution of the empirical p-vales, which is consistent with these 17 variables having the same means jointly for urban versus rural CP cases. In subsequent columns, we add additional covariates, concluding with 52 covariates in Column 4. In each case, we failed to reject the null of no difference in all covariate means jointly for urban versus rural CP cases. We present results from a linear probability model estimated by Ordinary Least Squares (OLS) in Table 3. This simplest possible estimation technique would not provide consistent estimates of the causal effect of the level of care on the ambulatory status of children with CP if indeed the level of care is not randomly assigned.^3^ With this caveat, we did not find evidence that the level of care affects the CP ambulatory status using OLS. The coefficient on the first line of Table 3, − 0.0349, is not significantly different from zero.

**Table 2:** Resampling

	Specification 1	Specification 2	Specification 3	Specification 4
Empirical p-values (means and standard deviation)	0.491 (0.272)	0.498 (0.230)	0.510 (0.246)	0.52 (0.245)
Kolmogorov-Smirnov test p-value	0.641	0.273	0.401	0.179

**Specification 1** (17 variables) includes the following variables: number of pregnancies, number of births known, number of still births, number of abortions, number of miscarriages, number of premature children known, fertility treatments used, dummy if the child had presented with convulsions, sex of the child, dummy for in vitro fertilization

**Specification 2** (38 variables) includes additionally the following variables: preeclampsia, eclampsia, presence of chorioamnionitis, resuscitation at birth indicator, bleeding during the first trimester, bleeding during the second semester, bleeding during the third trimester, alcohol during pregnancy, tobacco during pregnancy, hypertension gestational diabetes, trauma during pregnancy, drugs during pregnancy, number of premature children known, CP family history, gestational hypertension, intrapartum asphyxia, neonatal encephalopathy, cooling, encephalopathy

**Specification 3** (50 variables) includes additionally the following variables: non-ambulatory CP status, presence of post-neonatal cause, type of delivery, number of gestation weeks, head circumference, birthweight, number of hospitalization days during pregnancy, Apgar score at 1 min, Apgar score at 5 min, Apgar score at 10 min, last recorded head circumference, age at the initial registration

**Specification 4** (56 variables) includes additionally the following variables: mother’s age at time of birth, dummy for single pregnancy, prescription drugs indicator, severe illness during pregnancy, gross motor function classification of CP severity, severity of encephalopathy, non-ambulatory CP status, presence of post-neonatal cause, type of delivery, number of gestation weeks, head circumference, birthweight, number of hospitalization days during pregnancy, Apgar score at 1 min, Apgar score at 5 min, Apgar score at 10 min, last recorded head circumference, age at the initial registration

**Table 3:** Linear Probability Model Results^a^

VARIABLES	Linear regression
Level III or II vs Level I	−0.0349 [0.047]
Asphyxia	0.2647** [0.071]
Preeclampsia	0.1597+ [0.095]
Blood during the first trimester	0.0912+ [0.049]
Severe illness during pregnancy	−0.0152 [0.050]
Preterm birth	0.1860* [0.079]
Birthweight	0.0001 [0.000]
Years of maternal education	−0.0001 [0.012]
Mother’s age at time of birth	0.0040 [0.004]
Vaginal delivery	0.0514 [0.124]
Vaginal delivery after cesarean	−0.1369** [0.043]
Drugs	0.1495 [0.136]
Hypertension	0.1630* [0.074]
Trauma	−0.0081 [0.059]
Family CP history	−0.1053 [0.088]
Observations	825
R-squared	0.08

*** *p* < 0.01, ** *p* < 0.05, *p < 0.1

^a^Level I hospitals are the base category. Robust standard errors in brackets

In Tables 4, 5 and 6, we estimated the effects of level of care using the instrument of the mother’s residence at birth to correct for endogeneity of treatment level. Using three different estimation techniques (2-Step GMM, Endogenous Bivariate Probit, and 2SLS), we were again unable to find significant evidence that the level of care available at time of birth impacts the later CP non-ambulatory status.

**Table 4:** Instrumental Variables Results (Level III&II vs Level I)

LEVELS	2-Step GMM	Endogenous bivariate probit
III&II vs I	0.006 [0.128]	0.01 [0.03]
Observations	825	825
Overid	0.281	
Overid P-val	0.87	
F-test	47.9	
Endog	0.02	
Endog P-val	0.87	
Rho		−0.08 [0.258]
Chi-sq		0.001
F-analog		65,341
Exon P-val		0.914

*** *p* < 0.01, ** *p* < 0.05

^a^Table presents reduced form estimates of the effects of interest from two stage least squares, generalized method of moments, endogenous bivariate probit. Robust standard errors in brackets. *** *p < 0.01*, ** *p < 0.05.* Over is the Hansen J test for overidentification; Endog is a test for endogeneity of exposure variable; F-test is Angrist-Pischke multivariate F-test of excluded instruments; Chi-sq is Wald test of Rho = 0. F-analog is the test of joint significance of instrumental variables. Exon P-val is the smallest *p-value* of the excluded instrument in the regression of residuals on covariates and instrumental variables. 2SLS estimates are numerically equivalent 2-Step GMM up to two decimal places

**Table 5:** First stage complete results (Level II & III vs Level I)^a^

Variables	
Asphyxia	0.0452 (0.0700)
Preeclampsia	0.0389 (0.0817)
Blood during the first trimester	0.000762 (0.0553)
Severe illness during pregnancy	−0.0200 (0.0510)
Preterm birth	0.124 (0.0810)
Birthweight	−4.64e-05 (3.88e-05)
Years of maternal education	0.0151 (0.0116)
Mother’s age at time of birth	0.00419 (0.00403)
Vaginal delivery	−0.0232 (0.128)
Vaginal delivery after cesarean	0.0471 (0.0461)
Drugs	0.0874 (0.116)
Hypertension	−0.0116 (0.0705)
Trauma	0.0127 (0.0566)
Family CP history	0.00681 (0.0965)
Montreal dummy	0.142*** (0.0485)
Gatineau dummy	0.390*** (0.0509)
Quebec dummy	−0.291*** (0.0799)
Sherbrooke dummy	0.366***
Observations	825

** *p* < 0.01, * *p* < 0.05, + *p* < 0.1

^a^Robust standard errors in brackets. Centered R-squared = 0.31

**Table 6:** Second stage complete results (Level II & III vs Level I)^a^

VARIABLES	
Level III or II vs Level I	−0.0565
	[0.129]
Intrapartum Asphyxia	0.2676**
	[0.070]
Preeclampsia	0.1594+
	[0.093]
Blood during the first trimester	0.0935+
	[0.049]
Severe illness during pregnancy	−0.0145
	[0.049]
Preterm birth	0.1884*
	[0.077]
Birthweight	0.0000
	[0.000]
Years of maternal education	0.0014
	[0.012]
Mother’s age at time of birth	0.0039
	[0.004]
Vaginal delivery	0.0507
	[0.121]
Vaginal delivery after cesarean	−0.1342**
	[0.042]
Drugs	0.1469
	[0.135]
Hypertension	0.1629*
	[0.072]
Trauma	−0.008*
	[0.057]
Family CP history	−0.102
	[0.08]
Observations	825

** *p* < 0.01, * *p* < 0.05, + *p* < 0.1

^a^Robust standard errors in brackets. Centered R-squared = 0.31

In the first column of Table 4, we included a number of test statistics assessing the quality of our models. We report *p*-values from the Hansen J test for overidentifying restrictions, the Anderson-Rubin Wald test of joint significance of the program effects that is robust to weak identification, as well as the Angrist-Pischke multivariate F-test of excluded instruments. We found that our instruments were not weak (F-statistic of 47.9 > 10), and that the model is not overidentified. However, the test of endogeneity did not reject the null hypothesis that treatment variable can be treated as an exogenous regressor.

We present the estimated causal effect of level of care on ambulatory status using an endogenous bivariate probit model in Column 2 of Table 4. The standard error of the estimated effect [0.03] is dramatically lower than for the other 2 instrumental variables techniques, indicating a more precise estimation of any effect. Here again, we did not find any significant effect of the level of care on ambulatory status. Tables 5 and 6 present the first and second stages of 2-Stage Least Squares (2SLS) estimates of treatment effects. The coefficient of interest, as seen on the first line of Table 6, again did not significantly differ from zero.

## Cost-benefit analysis

Neonatal care in Canada and around the world is extremely expensive. Healthcare systems aim to find optimal ways to deliver increasingly expensive neonatal care. For example, Gavurova et al. show that in Slovakia day surgeries related to Gynaecology and Obstetrics had increased significantly after this form of healthcare provision was regulated in 2004 [45]. In Canada the cost of treatment in an ICU and the cost per general ward varies by the type of facility. For example, in British Columbia according to 2014/2015 Interprovincial reciprocal billing rates, the cost of an ICU per day varies from $1949 to $5317 and the cost for a general ward per day varies from $803 to $2794 per day l [46]. We estimate the savings related to hospitalization costs only from a hypothetical alternative assignment of births at risk of CP to maternity facilities rather than the higher level of care facilities currently observed in the data.

For our cost-benefit analysis we used a 3.8 per 1000 live births incidence rate of CP and a projected number of 391,414 births for 2016 in Canada. It is expected that 1523 children with CP will be born in Canada in 2016. For our computations we also used the lowest rates from the literature of $1628.60 per ICU day and $388 per ward [47].

Our data contains the number of hospital days for each patient, stratified into general ward and intensive care unit. The median hospitalization days for CP children born in a level I hospital is 6 days. The median number of hospitalization days for CP children born in level II, or level III care is 28.5 days, and the difference in hospitalization days between two types of hospitals is 23 days (95% CI, 17.06–28.94) per patient. A little over 20% of CP children born in level I hospital are subsequently transferred to level II or level III hospitals.

Under these assumptions the total cost related to hospitalization days given current matching of CP births to maternity facilities is $49,875,355. However, if CP related births were assigned to nurseries and transferred subsequently to hospitals with NICU, the overall total hospitalization costs would decline to a little over $34 million per year, saving the Canadian healthcare system around $15,865,600 (95% CI: $6,131,184 - $24,103,478). Our estimates suggest that significant hospitalization costs might be averted if pregnant women at risk of having a CP child would give birth in level I care and then be transported to a level II, or Level III after birth if necessary. One limitation of our cost-benefit analysis is that mothers at risk of having a CP child are not identified in advance. However, this is a lower bound of savings given that many direct healthcare costs were excluded from analysis.

## Discussion

We have shown that differences in the level of neonatal care, and associated medical technology available at the time of birth, do not significantly affect the risk of CP non-ambulatory status. This finding is consistent and robust across methods based on selection on observable assumption as well as using methods based on selection on unobservables documented in this paper. Instrumental variables estimation allowed us to control for possible unobserved selection effects and we were not able to find a significant relationship between the level of neonatal care at birth hospital and CP non-ambulatory status across all estimated models. Resampling analysis revealed that the distribution of clinical, social and economic covariates appears to be consistent with random reassignment with respect to location at birth (rural vs urban). Our cost-benefit analysis suggests that around $16 million per year in healthcare savings could accrue to the Canadian Healthcare system if CP related births were assigned to nurseries and subsequently transferred to level II, or III hospitals if necessary.

The lack of incremental impact of the level of neonatal care at the time of birth on the later risk of CP non-ambulatory status likely demonstrates the benefit of the development and generalization of the neonatal resuscitation program (NRP). The NRP educational program for North American healthcare providers working in the delivery rooms and nurseries is designed to aid in learning the cognitive and technical skills required for effective evidence-based resuscitation of newborn babies and appropriate referral to specialized centers as soon as possible [48, 49]. Neonatal resuscitation was shown to reduce mortality from intrapartum related events [26, 50, 51], such as perinatal asphyxia, and might explain the lack of effect found in our study.

Our study has several limitations. The methods employed cannot replace a prospective randomized controlled trial (RCT), and may not have fully controlled for selection effects or unobserved covariates. However, a RCT that would assess the impact of levels of neonatal care available at hospitals where delivery was carried out on CP ambulatory status is unlikely to be implemented given pragmatic considerations and concerns. Our study does not discuss the differences in other measures that might be important as well for determination of CP related outcomes of regionalized perinatal care such as fine motor skills, cognition, language, or behavior.

We used maternal residence at birth as an instrument to predict the type of hospital at birth. This may not always be a perfect predictor for the level of neonatal care at delivery hospital especially in large metropolitan areas such as Montreal for example, where the pregnant women can choose either level of NICU. However, geographic accessibility is one of the most significant determinants of choice of maternity care providers [36, 37]. Given the peculiarities of a universal healthcare system ceteris paribus, a pregnant woman is more likely to choose a delivery hospital with the highest available level of care.

## Conclusion

Our study provides empirical evidence that the level of care available at birth does not significantly affect the distribution of CP non-ambulatory cases. The success of the neonatal resuscitation program may contribute to this lack of relationship. This does not mean that neonatal care is not effective, as the overall impact of neonatal interventions on health outcomes is well documented in the health services and economics literature [7–9, 52]. However, our results indicate the potential for significant healthcare related savings within the existing referral process to birthing centers and overall neonatal care rationing.

An equitable and affordable healthcare system requires a constant search for the optimal delivery of increasingly expensive neonatal care. In this study, we have identified that the existing matching mechanism of births to hospitals within large Canadian metropolitan areas could be improved. This means that policy makers could design better triage policies and increase the productivity of limited neonatal healthcare resources’.

## Data Availability

The datasets generated and/or analyzed during the current study are available in the Cerebral Palsy Registry https://www.cpregistry.ca/

## Appendix

### Definitions of the levels of the neonatal care used in Quebec

1A: basic care + phototherapy

1B: greater or equal to 34 weeks of gestation, intravenous therapy, gavage feeding

2A: greater or equal to 32 weeks of gestation, intravenous therapy, gavage feeding

2B: greater or equal to 32 weeks of gestation, intravenous therapy, gavage feeding and ventilation via nasal passage

2B+: greater or equal to 30 weeks of gestation, intravenous therapy, gavage feeding and ventilation via nasal passage or endotracheal ventilation

3A-: greater or equal to 29 weeks of gestation, endotracheal ventilation + NO. Immediate access to all specialists.

3A: care provided to all babies regardless of their gestational age or birth weight. Endotracheal ventilation + NO. Immediate access to all specialists.

3B: level 3A care and complete access to specialists. Imaging tests carried out and interpretation of results done. Surgeries done except for severe cardiac malformations requiring extracorporeal circulation.

3C: Level 3B care + surgical repair of severe cardiac malformations requiring extracorporeal circulation.

## Abbreviations

CP: Cerebral Palsy
NICU: Neonatal Intensive Care Unit
CCPR: Canadian Multi-Regional Cerebral Palsy Registry
CDC: The US Center for Disease Control
NE: Neonatal Encephalopathy
GMFCS: Gross Motor Function Classification System
2SLS: 2-Stage Least Squares
NRP: Neonatal Resuscitation Program
RCT: Randomized Controlled Trial

## Footnotes

1 Hospitals in Quebec are not-profit entities. As non-for profit organizations, public hospitals organizations are expected to be efficiently use limited healthcare resources [39].

2 To instrument for hospital intensity we also used physical distance between mother’s residence at birth and closest high intensity hospital. We used Google’s Distance Matrix API. Our results did not change.

3 Since the level of care can be influenced by self-selection, it would by definition be non-random and require estimation using exogenous instruments. However, statistical tests reported in Table 4 fail to find evidence of non-random assignment.
